# A generative adversarial network-based accurate masked face recognition model using dual scale adaptive efficient attention network

**DOI:** 10.1038/s41598-025-02144-2

**Published:** 2025-05-21

**Authors:** Jafar A. Alzubi, Kiran Sree Pokkuluri, Rajesh Arunachalam, Surendra Kumar Shukla, Sumanth Venugopal, Karthikayen Arunachalam

**Affiliations:** 1 Faculty of Engineering, Al-Balqa Applied University, Salt, 19117 Jordan; 2 Department of Computer Science and Engineering, Shri Vishnu Engineering College for Women, Bhimavaram, Andhra Pradesh 534202 India; 3 Department of Electronics and Communication Engineering, Saveetha School of Engineering, Saveetha Institute of Medical and Technical Sciences, Thandalam, Chennai, Tamil Nadu 602105 India; 4 Department of Computer Science and Engineering , Amity School of Engineering and Technology, Amity University, Noida, Uttar Pradesh India; 5 Department of Information Technology, Manipal Institute of Technology Bengaluru, Manipal Academy of Higher Education, Manipal, Karnataka 576104 India; 6 Department of Electronics and Communication Engineering, P.T. Lee Chengalvaraya Naicker College of Engineering and Technology, Oovery, Kanchipuram, Tamil Nadu, India

**Keywords:** Masked face recognition, Dual scale adaptive efficient attention network, Generative adversarial network, Enhanced addax optimization algorithm, Computational biology and bioinformatics, Environmental social sciences, Medical research, Engineering

## Abstract

Masked identification of faces is necessary for authentication purposes. Face masks are frequently utilized in a wide range of professions and sectors including public safety, health care, schooling, catering services, production, sales, and shipping. In order to solve this issue and provide precise identification and verification in masked events, masked facial recognition equipment has emerged as a key innovation. Although facial recognition is a popular and affordable biometric security solution, it has several difficulties in correctly detecting people who are wearing masks. As a result, a reliable method for identifying the masked faces is required. In this developed model, a deep learning-assisted masked face identification framework is developed to accurately recognize the person’s identity for security concerns. At first, the input images are aggregated from standard datasets. From the database, both the masked face images and mask-free images are used for training the Generative Adversarial Network (GAN) model. Then, the collected input images are given to the GAN technique. If the input is a masked face image, then the GAN model generates a mask-free face image and it is considered as feature set 1. If the input is a mask-free image, then the GAN model generates a masked face image and these images are considered as feature set 2. If the input images contain both masked and mask-free images, then it is directly given to Dual Scale Adaptive Efficient Attention Network (DS-AEAN). Otherwise, generated feature set 1 and feature set 2 are given to the DS-AEAN for recognizing the faces to ensure the person’s identity. The effectiveness of this model is further maximized using the Enhanced Addax Optimization Algorithm (EAOA). This model is helpful for a precise biometric verification process. The outcomes of the designed masked face recognition model are evaluated with the existing models to check its capability.

## Introduction

Facial recognition is a process of identifying the features of a masked region in a face that requires prior information about the particular person. The artificial vision research field is attracted to the current study scenario of occluded face recognition^1^. The goal of earlier occluded face identification systems is to recognize and detect a person’s face, even if the masked portion of the face was irregularly positioned and shaped^2^. Meanwhile, the area around the lips, nose, and cheeks is frequently hidden by a mask. The forehead, eyebrows, and eyes are the remaining areas free of obstruction^3^. Consequently, a masked facial recognition tool might concentrate on analyzing features that are taken from the portions of the face, such as the forehead, eyes, and eyebrows^4^. The majority of terrorists and criminals wore masks to hide their identities^5^. Sunglasses, hats, and coloured festoons are served as masks. The essential features needed to identify a person are reduced by using these kinds of masks^6^. Compared to other standard face recognition techniques, masked face identification offers fewer challenges due to its reduced amount of face features^7^. As a result, the recognition accuracy rate is declining based on the low quality of data and the varying environmental conditions.

Face recognition methods are extensively used in public spaces for verification of identity and access control since it is non-contact, extremely effective, and user-friendly^8^. When a mask covers a significant portion of the face, then face recognition gets harder. Consequently, it is crucial to explore how facial masks affect face recognition system behaviour and create mitigation strategies to reduce performance loss^9^. The branch of periocular-based authentication is very useful for facial feature identification and significant progress in recent years. It is demonstrated that one of the most vital and sensitive locations is a periocular region and this region is utilized successfully for biometric identification^10^. Typically, the region used in periocular biometrics is the face area surrounding the eyes^11^. In some cases, identifying the face is difficult based on occlusion or masks, such as the usage of hats, scarves, sunglasses, and disguise elements in makeup. These kinds of masks reduce the success rate of recognition of face features^12^.

The rise of large-scale datasets and sophisticated Convolution Neural Networks (CNN) resulted in the formation of deep methods in face recognition tasks^13^. The deep features always show inadequate invariance due to a mask, where the entire facial image cannot be applied for description, even though deep learning models have shown success in general face recognition settings^14^. Consequently, using face masks presents a big research challenge. Initially, a large-scale training dataset comprising various sorts of mask-wearing faces must be collected. Creating a training dataset of this scale requires a significant investment of time and money in labor, but it also takes time to keep the variety of the data in these datasets^15^. Therefore, there is an urgent need for an affordable and practical face data augmentation approach. Second, it is crucial to reduce the efficiency loss from the design of the framework in accordance with face mask characteristics. The most popular approaches in the literature for masked face recognition are CNN and transfer learning of previously trained deep learning models^16^. Yet, SVM requires a lot of training time. The most concerning drawbacks of transfer learning are overfitting and negative transfer^17^. Additionally, masked identification of faces is particularly difficult because there are very few reliable labelled datasets. Due to these limitations, an effective deep network-based masked face recognition model is developed.

The main contributions of the developed framework are given in the below points.

- To develop a new face recognition model that successfully identifies masked faces with higher quality. This developed model is used for user identification in smartphones, computers, and tablets, making it a quick and safe way to unlock devices and access personal data.
- To implement GAN-based image augmentation that increases the capacity of models by allowing the trained model to become more resistant to changing conditions and input data, which results in more precise predictions and adaptability on previously unidentified information.
- To develop an EAOA-based optimization strategy that improves the network’s overall success rate by fine-tuning parameters, modifying learning rates, and refining the network topology to obtain higher levels of precision and effectiveness while recognizing the masked faces.
- To design a DS-AEAN-based identification phase that offers a single model for masked face recognition, providing an extensive solution to the issues of recognizing people wearing masks.
- The designed masked facial recognition framework is assessed using a variety of metrics to determine its efficacy and consistency.

Overall view of the implemented framework is offered in the below sections. A literature review of existing work along with the benefits and features of existing techniques are detailed in “Literature Survey” section. The architecture view of the proposed model and the description of datasets are provided in “Development of a Masked Face Recognition Model using an Attention-based Deep Network with its Image Collection Representation” section. The image augmentation mechanism and optimal tuning of parameters using the proposed algorithm are described in “Description of Image Augmentation and Optimal Tuning of Parameters using the proposed Optimization Strategy” section. The description of the attention-based network for accurate mask recognition is given in “Brief Elucidation of Implemented Attention-based Deep Network for Accurate Masked Face Recognition section. Experimental analysis and the summary of this proposed model are generated in “Results and Discussions” and “Conclusion” sections, respectively.

## Literature survey

This section provides a detailed review of existing masked face recognition with deep and machine learning techniques. Here, “Related works” section displays the classical literature works regarding masked face recognition and also observes different techniques used for the recognition of masked faces. Moreover, common issues presented while designing a novel masked face recognition model are detailed in “Problem statement” section. Various advantages and limitations of existing literature works are tabulated in Table 1, which provides a great contribution in designing a novel framework with higher accuracy.

**Table 1:** Advancements and complications of classical masked face recognition techniques.

Author [citation]	Techniques	Advancements	Difficulties
Sumathy et al.^18^	Double Generator Network	It accurately recognizes the face behind the mask by splitting the input images into higher and lower frequency attributes	This approach suffers from shift invariance problems
Kocacinar et al.^19^	CNN	It does not require plenty of samples for performing masked face recognition It identifies the incorrect usage of masks in the face	It does not rectify the Interpretability challenges and Computational complexity issues
Zhang et al.^20^	AMaskNet	It utilizes a contribution estimator and feature extractor to detect masked faces at low cost It performs simple matrix multiplications to refine the feature representation of a face	The generalization capacity of the model is low and gradient issues are high
Ullah et al.^21^	DeepMaskNet	It prevents the overfitting issues during the classification phase by using a dropout technique It retrieves deep, discriminative, and descriptive features for recognizing the masked faces	Labeled data requirement is high
Faruque et al.^22^	CNN	It employed an operational efficiency depth-wise normalization and batch normalization to progress the operational efficiency of masked face detection	The performance of the framework decreases with respect to the increase in data The superiority of the model is low when there is variation or changes in face angles
Golwalkar and Mehendale^23^	FaceMaskNet-21	It has the ability to detect masked faces in static video files, live video streams, and static images	It does not handle occlusions in images This model struggles to detect the masked face when the face angle is different in the input frame
Vu et al.^24^	CNN	It has high feasibility in retrieving facial features It retrieves only the relevant features to obtain accurate recognition outcomes	The energy consumption of this network is high due to the usage of more parameters
Eman et al.^25^	RPCA and MobileNetV2	It analyzes the key facial features to find the location and presence of masks on a face It solves the occlusion issues	Accuracy is low while detecting faces in images with bad lighting conditions Different kinds of occlusions like hats and scarves cannot be removed in this model

### Related works

In 2024, Sumathy et al.^18^ have presented a unique double generator network based on neural networks to accurately detect the face hidden behind the mask images. First, the acquired images were divided into lower and high-energy components. The masked image attributes were then captured and the individual faces were recognized through the Haar cascade classifier. Edge synthesis and image reconstruction were the two modules that made the suggested double generator networks more powerful. Dilated CNN was used in the first modules to extract appropriate data from masked facial images. In the next section, the created edges were reflected and used to recreate the original edges. This innovative model outperformed better than previous frameworks in terms of quality.

In 2022, Kocacinar et al*.*^19^ have presented a model to generate a masked face dataset in order to recognize people who either do not wear masks or wear them inaccurately and to validate their identities. Based on this, a brand-new face recognition smartphone application and actual time-masked identification services were created using an ensemble of optimized neural networks. Tests conducted on the five datasets showed that the recommended method appreciably boosts the quality of masked detection of faces over other existing contemporary techniques.

In 2022, Zhang et al*.*^20^ have suggested a neural method to lessen the adverse impact of mask flaws on face recognition. In this model, an Attention-Aware Masked Face Recognition (AMaskNet) was made to boost masked face detection ability. It consists of two sections, a contribution predictor and a tool for extracting features. The contribution predictor was utilized to determine each attribute that enhanced the feature representation. In the meantime, the entire model was optimized using the comprehensive training technique.

In 2022, Ullah et al.^21^ have proposed a unique DeepMasknet framework that could recognize faces when masked. To assess the success rate of detecting masks and masked facial identification techniques, this proposed scheme generated an extensive and varied mask identification dataset. Experiments conducted on several datasets, especially a cross-dataset setup demonstrated the DeepMasknet framework being superior to current models.

In 2024, Faruque et al*.*^22^ have proposed a CNN model intended for identifying the masked identity more accurately. In order to optimize the overall performance, batch normalizing, dropout, and depth-wise normalization were incorporated to fulfil specific requirements and improve operational efficiency. These methods reduce overall complexity while increasing the model’s reliability and productivity. This offered approach provides a viable and effective approach to identify masked faces in everyday situations based on the demands of the task and rigorously refining the model architecture.

In 2022, Golwalkar and Mehendale^23^ have proposed a deep metric model for identifying the masked image for authentication purposes. This system could be used to identify individuals in CCTV footage from locations such as shopping centres, banks, and ATMs. Due to its quick performance and precision, this system could be used to track the presence of unauthorized users in banks, schools, colleges, and other high-security areas.

In 2022, Vu et al*.*^24^ have offered a novel method for reliably recognizing masked faces by combining LBP textual pattern extraction with deep deep-learned networks. This novel approach advanced biometric verification and authentication systems under harsh environmental settings by providing a viable means of reliable and precise masked identification of faces in practical situations.

In 2023, Eman et al.^25^ have proposed a novel hybrid approach that combines Robust Principle Component Analysis (RPCA) for successful masked identification of faces. This method divides an image into occluded and non-occluded components using RPCA and pre-trained using the MobileNetV2 network for mask recognition. The suggested approach outperformed better than previous techniques in terms of proficiency and resilience to occlusion, achieving high masked face recognition.

In 2023, Alqaralleh et al.^26^ have proposed a novel framework for recognizing the masked face from profile and frontal faces. The suggested technique framework used the binarized statistical features to obtain the texture-based descriptors. Moreover, CNN was employed to acquire the significant features and perform masked face recognition. Further, various experiments were executed to compute the reliability and performance of the implemented masked face recognition model.

In 2023, Hsu et al.^27^ have proposed a novel framework for recognizing the masked faces of individuals with deep learning techniques. Here, required facial images were sourced from benchmark resources and provided to the training phase. Later, the feature embedding procedure was executed by ResNet-100. Moreover, the developed framework considered various loss functions to execute the verification and identification procedures. Validation outcomes displayed that the recommended technique accomplished superior outcomes than classical models.

### Problem statement

Face recognition is one of the biometric validation approaches. Many organizations use this approach to recognize individuals. Face recognition systems are useful in airports, railway stations, and many other public places. However, recognizing people with their masks on is a difficult process. Therefore, many research works have been published to provide new ideas or techniques for recognizing masked face images. Yet, the existing strategies are not reliable due to some challenges and they are listed below:

- The recognition rate of the traditional models is low as they are not capable of detecting faces in low-quality and noisy images.
- Some of the conventional techniques perform image normalizing, de-noising, sharpening, resizing, and cropping processes. But, performing all these steps takes a lot of time.
- Plenty of samples are needed in the conventional techniques to obtain a high detection rate.
- Most of the existing techniques are not capable of handling different kinds of occlusions during the recognition process of a masked face.
- Some of the models are trained with only masked face images and some others are with mask-free images. Recognizing the faces by considering both options is complex.

Therefore, an advanced deep learning approach will be developed to rectify these issues and identify the masked faces with high accuracy. Table 1 provides the features and drawbacks of existing masked face recognition models using deep learning.

## Development of a masked face recognition model using an attention-based deep network with its image collection representation

This phase discusses about the need for recognizing the masked face and also several complications faced by the classical techniques in different fields are represented in “Need for masked face recognition” section. Furthermore, a detailed discussion about the newly designed masked face recognition model is offered in “Block schematic view of developed masked face recognition model” section with structural representation. Moreover, the dataset employed for the validation and also their deep details like class name, image count and image sizes are discussed in “Masked face images description” section with sample images.

### Need for masked face recognition

Usage of face masks during the COVID-19 pandemic has highlighted the consequence of masked face recognition. Traditional facial recognition techniques are intended for exposed faces and may struggle to reliably detect people wearing masks. As a result, there is an increasing demand for reliable facial recognition systems that will recognize people even while they are wearing masks.

Masked face recognition used in various fields and their needs are provided in the below points.

- *Public Safety* To maintain public safety and security, every security checkpoint, public transportation hub, and other public venues must reliably recognize the individuals, even those wearing masks.
- *Medical Settings* Masked face recognition is useful in the identification of medical personnel and patients while retaining processes for preventing infections.
- *Authentication* With the growing usage of facial recognition for authentication, it is critical to have systems that are capable of recognizing individuals wearing masks in order to maintain secure access to equipment and facilities.
- *Crime Prevention* Masked face recognition is able to assist law enforcement in identifying criminals even when they are wearing masks to hide their identities.

Thus, by creating reliable and precise masked face recognition systems, the reliability of security measures, and public health procedures, and ensuring quick authentication processes in masked environments are upgraded.

### Block schematic view of developed masked face recognition model

**Fig. 1:**
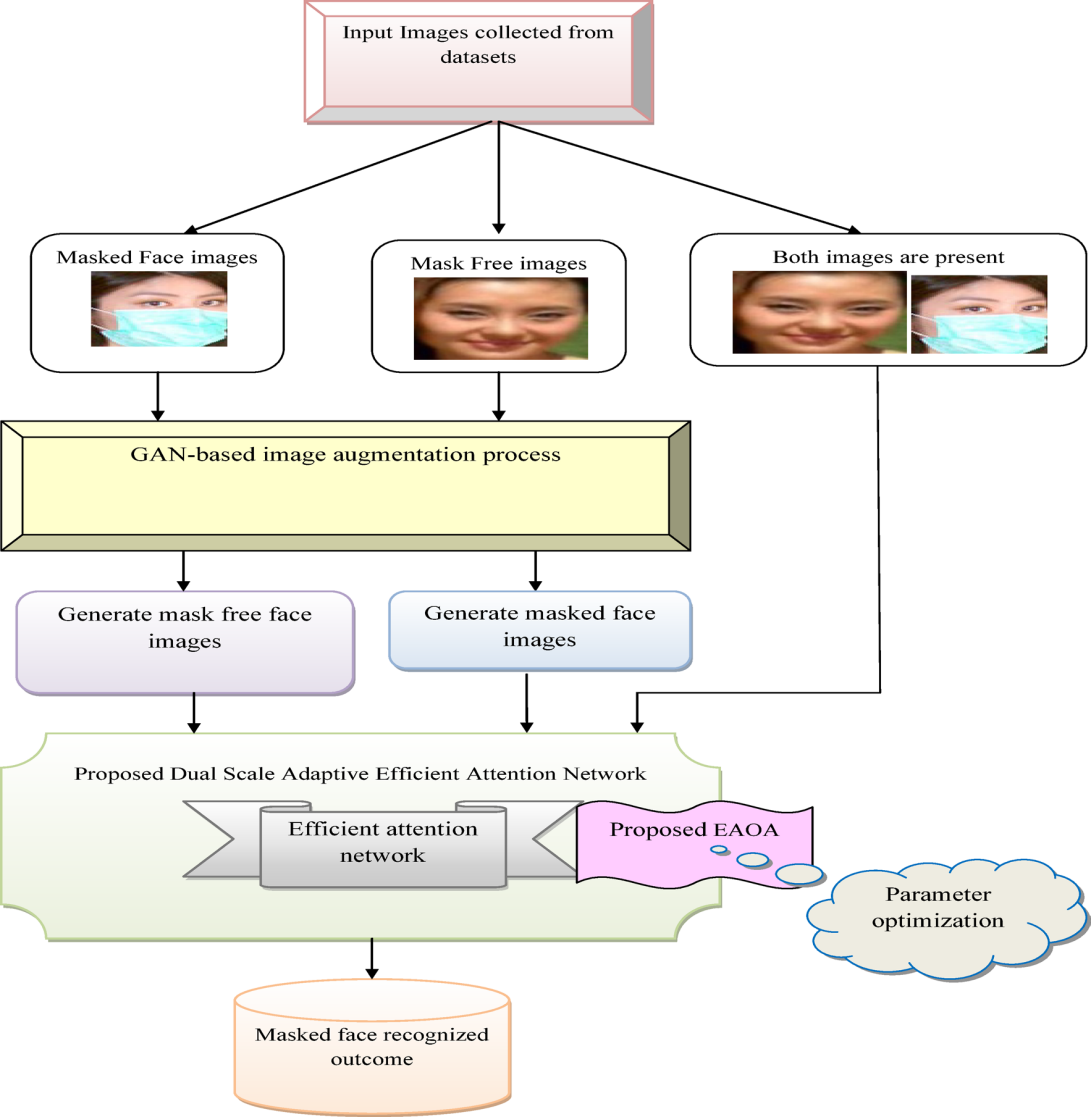
General architecture of deep learning-based masked face recognition technique.

An efficient masked face recognition model is employed to identify individuals even when they wear masks, which has become increasingly important in the context of public health measures and security protocols. The designed model helps to overcome the challenges due to partial face occlusion, which hinders traditional face recognition systems. Facial features like mouth and nose are necessary for the identification of the face, but in a masked face, these features are covered with a mask. Hence face recognition is a challenging process in masked faces. The limited image pixels in small face images make it difficult to remove a facial feature and decrease the precision of the face identification system. Low resolution and poor image quality are certain complications in the identification process. Extracting input data for water purity prediction is complex and expensive. It is difficult to acquire spatial features from the data. Hence, to overcome these difficulties a GAN-aided masked face recognition framework is implemented and its architecture is provided in Fig. 1.

An accurate masked face recognition model is designed to provide reliable and efficient authentication of individuals based on their facial features. This system is used for security purposes to ensure that authorized individuals are granted access. The raw images including both mask and without mask images are gathered from the required database and applied for augmenting the images. It expands the training dataset and boosts the model’s robustness to prevent overfitting issues. The GAN network is designed to perform the image argumentation process. By augmenting the training data with GAN-generated images, the designed framework extracts more robust features and boosts their performance in the classification phase. GANs generate high-quality synthetic images with mask f1 and without mask f2 that resemble real data, providing diverse and realistic samples for training. The augmented image from GAN is applied to the final classification phase. If the input images contain both masked and mask-free images, then it is directly given to DS-AEAN for categorization purposes. Here, the classification is carried out using the DS-AEAN structure. Dual-scale networks capture the input at multiple scales and capture high-level contextual information to upgrade the robustness of the categorization phase. During the testing period, if both images are not present then the image with a mask is applied to 1st scale of convolution and the image without a mask is applied to 2nd scale of convolution. Dual-scale networks help in localizing objects within an image more precisely by combining information from different scales. The success rate of the implemented mechanism is upgraded by fine-tuning the attributes like epoch count, hidden neuron count, and step per epoch count in an efficient attention network by EAOA to enhance the accuracy of the masked face recognition system. Based on the analysis results, the purpose of an accurate face recognition model is to enhance security and efficiency in different domains.

### Masked face images description

The designed model is trained, tested, and evaluated on a variety of datasets for face mask detection and masked facial identification. The descriptions of datasets are mentioned as follows.

Dataset 1(“Face Mask Detection Dataset”): The masked face images required for this model are collected from the link “https://www.kaggle.com/datasets/omkargurav/face-mask-dataset” accessed on 2024-06-28. In the current trend of global lockdowns related to the COVID-19 outbreak, face masks are required for everyone while travelling outside. Here, the model is trained to recognize face masks on 7553 images with three colour channels.

Dataset 2(“Real-World-Masked-Face-Dataset”): Another dataset containing images of people wearing masks and without masks is utilized to assess the model’s abilities for face mask detection under a variety of situations. The link of the second dataset is “https://github.com/X-zhangyang/Real-World-Masked-Face-Dataset/tree/master” accessed on 2024-06-27. The analysis is trained using the real-world database, which comprises several million tagged images from various categories. The previously trained versions of these models have been optimized for masked facial recognition.

**Fig. 2:**
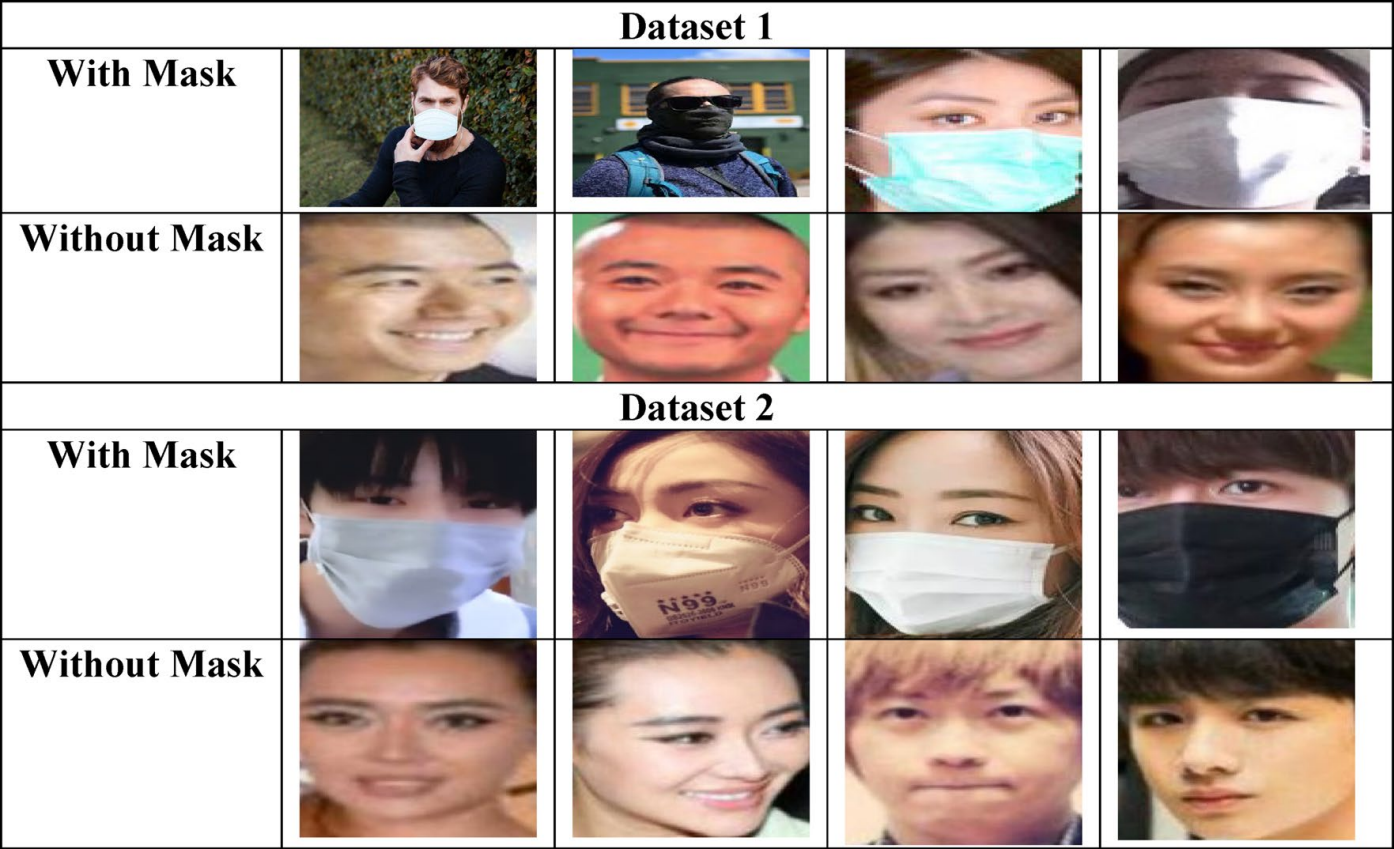
Sample images collected from the dataset.

The collected image is specified as $Is_{j}$, where $j$ is represented as $j = 1,2,3...J$. Here, the entire samples collected are specified as $J$. Image samples from datasets are provided in Fig. 2.

## Description of image augmentation and optimal tuning of parameters using the proposed optimization strategy

This section deals with the image augmentation procedures and also the novel optimization technique employed for tuning the essential parameters in the masked face recognition model. In “Generative adversarial networks” section, basic information about the GAN model is provided. “Image augmentation using GAN” section details the data augmentation process using GAN. Moreover, “Developed EAOA” section discusses about several modifications executed in newly designed optimization techniques and also their pseudocode.

### Generative adversarial networks

In this designed masked face recognition model, image augmentation is performed using GAN^28^ for generating high-quality synthetic images with mask (f1) and without masks (f2). These generated images are applied to DS-AEAN for classification. It is an artificial intelligence mechanism to generate unreal face images. Generator and discriminator are the two main neural networks used in GAN.

Generator: It is responsible for creating synthetic face images without the mask object. The generator is trained to complete the facial region where the mask is present, ensuring that the generated output is natural and reliable with the rest of the face structure. The loss functions help the generator to produce high-quality outputs that match the ground truth images. The loss function for generator training is specified in Eq. (1).

1 $$G_{lss} = \xi_{fc} \left( {R_{lss} + P_{lss} } \right)$$

Here, the term $R_{lss}$ and $P_{lss}$ are indicated as reconstructed loss and perceptual loss, respectively. The ground truth and generated image pixels varied and it is evaluated using Eq. (2).

2 $$L_{pxl} = \left| {E_{dit} - E_{gt} } \right|$$

Discriminators: It focuses on the entire face region and helps to implement the output created by the generator based on the original face image. The purpose of discriminators is to generate both semantically and visually consistent. The objective of the discriminator is provided in Eq. (3).

3 $$D_{lss} = \psi E_{gt} \log D_{rgn} \left( {E_{dit} ,E_{gt} } \right) + \psi E_{dit} los\left( {E_{dit} ,E_{gt} } \right)$$

By minimizing the objective function, the generator output is architecturally coherent with the original input face image. By training both the real and GAN-generated masked face images, the designed scheme extracts more robust features that are less sensitive to the presence of masks. Adversarial training helps the model to sustain the high reliability of the model even under challenging conditions such as varying lighting, occlusions, and different mask types. Even though, generating samples with GANs is computationally rigorous, especially for high-quality outputs. Real-time inference is challenging for some applications.

### Image augmentation using GAN

The raw input image $Is_{j}$ is applied to the image augmentation process. It is a method that uses the power of GAN to create realistic and distinct variations of existing images. GAN is trained in an aggressive way to produce high-quality synthetic data.

Generally, image augmentation procedures are widely employed in recognition, identification and classification models. Various deep learning-based and machine learning image augmentation techniques are used in different research work. ViT Huge-14^29^ is an image augmentation procedure, which is suitable for processing enormous image samples. The ViT Huge-14 use enormous parameters that easily subjected to overfitting issues and also it is highly depends on the global attention procedures for collecting the spatial relationships. Moreover, this system needs more training time to process the image samples, which may lead to enhance the implementation cost. Higher Order Dynamic Mode Decomposition (HODMD)^30^ is a kind of augmentation tool used in various applications to enrich the quality of information extracted from image samples. Yet, it technique includes complicated calculations to process the large data samples that aid in improving the overall expense. In some cases, these models are sensitive to noise which creates several difficulties in validating the higher dimensional images. In order to tackle several complications presented in the existing framework, the suggested masked face recognition model employed GAN. The GAN has the efficiency to tackle the overfitting issues by creating novel test samples to enhance the sample count in a dataset and then it learns the generalizable patterns to execute the procedures. Sample image patterns created by GAN are in higher quality and also it has the efficiency to identify various features in specific classes. The working patterns of GAN are quick and also process enormous samples in minimal time.

During the training phase, the GAN learns to produce new images similar to the original dataset. The generator modifies previous images by adding small disturbances, changes, or distortions. These created images are utilized to enrich the training dataset and increase its diversity. This improves the model’s scalability and resilience by exposing it to a broader variety of input data variances. GANs are capable of creating image variations such as changes in color, texture, position, lighting conditions, and other visual characteristics. This is able to generate a more diverse and robustly trained dataset for the predictive model.

Benefits of image augmentation using GAN.

- GANs are capable of producing diverse and realistic image variations, resulting in an expanded training dataset.
- Training on augmented data allows neural network models to better adapt to previously unknown data and variances in real-life situations.
- GAN-based augmentation is able to train the network faster by minimizing the need to collect and classify vast volumes of new data.
- GAN-based image augmentation is often used in computer vision, healthcare imaging, and language analysis to improve the performance of predictive models by providing a more diverse training dataset.

**Fig. 3:**
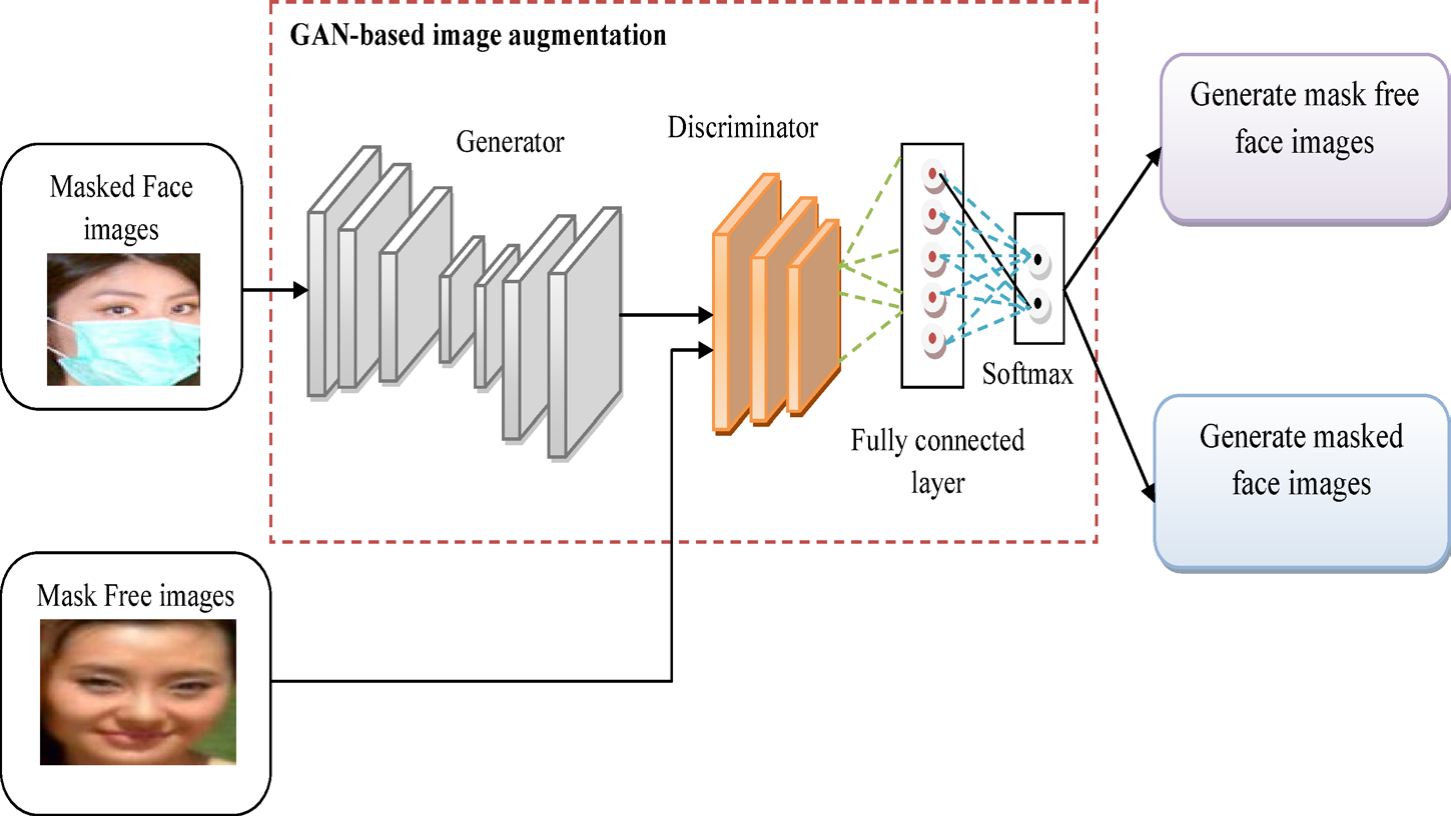
Pictorial representation of GAN-based image augmentation.

Using GANs for image augmentation strengthens the quality, variety, and interpretation capabilities of their predictive models, resulting in improved efficiency in tasks like image classification, and object recognition. Finally, the augmented image is provided as the outcome. The pictorial representation of the GAN-based augmenting image is provided in Fig. 3.

### Developed EAOA

EAOA has developed for an efficient masked face recognition model, which serves an important role in training deep learning models for masked facial recognition. It iteratively updates the DS-AEAN parameters including step per epoch count, epoch count, and hidden neuron count. These techniques attempt to reduce the loss function by modifying the model parameters to enhance the efficacy of training data. Conventional AOA assists the model in resolving to a set of ideal parameters that reduce the loss function and increase its ability to perform on previously unseen data. AOA strategies prevent overfitting issues by normalizing the model during training and prevent the model from storing noise in the training data to improve its generalization capacity, resulting in greater accuracy. Moreover, AOA particularly with a large number of variables is computationally expensive to train and a vast dataset is required for reliable optimization strategies. Hence, AOA is enhanced by upgrading the random variable $G$ in Eq. (5) based on Eq. (4). The numerical form of the upgraded concept is provided in Eq. (4).

4 $$G = \frac{{V_{k,l} }}{{\left( {Bst + wst + Mft} \right)}}$$

Here, the best, worst, and mean fitness values are indicated as $Bst$, $wst$, and $Mft$, respectively. The random variable is upgraded based on these fitness values. This modification helps to provide faster convergences and results in faster training processes and more effective model optimization.

AOA^31^: Addaxes are known for their ability to track rainfall and identify the region with abundant vegetation foraging. They feed on grasses, shrub leaves, leguminous plants, herbs, and bushes. Addaxes accomplish individual and extensive searches to spot the food sources. This algorithm starts with the initial population and the objective function is evaluated for each member of the population. This algorithm then proceeds with iterative optimization and upgrades the position of new members based on the foraging and digging simulations. The initial position of Addaxes is algebraically expressed using Eq. (5).

5 $$F_{k,l} = B_{lwr} + G \cdot \left( {B_{upr} - B_{lwr} } \right)$$

Here, the population matrix is indicated as $F_{k,l}$, $G$ is the random variable varies in the range of $\left[ {0,1} \right]$. The term $B_{upr}$ and $B_{lwr}$ is indicated as the upper and lower limits of the variable.

Foraging process: The foraging process in AOA helps to determine the possible regions that enclose optimal solutions. During the foraging phase, the spot of individuals in a group is updated based on the shifts observed in addaxes’ positions while foraging for food. The adjustments in population members locations during the foraging process are mathematically modeled to guide the exploration phase effectively using Eq. (6) and Eq. (7).

6 $$F_{k,l}^{m1} = F_{k,l} + G \cdot \left( {CA} \right) - V_{k,l} \cdot x_{ij}$$

7 $$F_{k,l}^{m1} = \left\{ {\begin{array}{*{20}c} {F_{k,l}^{m1} } & {P_{k,l}^{m1} \le F_{k}^{{}} } \\ {F_{l}^{{}} } & {else} \\ \end{array} } \right.$$

Here, the term $CA$ is indicated as the identified area for foraging, and $V_{k,l}$ is the current spot. The objective function value of $l^{th}$ iteration is indicated as $F_{k,l}^{m1}$. By upgrading new position, AOA boosts the recovery of optimal solution.

Digging phase: To progress the exploitation phase, which concentrates on nearby search management within the resolving issues space, the AOA design simulates the digging talent. Addaxes dig by making small positioning modifications while making indentations in the sand. The placements of the population members of the algorithm are adjusted in accordance with these slight positional shifts, which are mathematically described in Eq. (8) and Eq. (9).

8 $$F_{k,l}^{m2} = F_{k,l} + \left( {1 - 2G} \right) \cdot \frac{{B_{upr} - B_{lwr} }}{d}$$

9 $$F_{k,l}^{m2} = \left\{ {\begin{array}{*{20}c} {F_{k}^{m2} } & {P_{k,l}^{m2} \le F_{k}^{{}} } \\ {F_{l}^{{}} } & {else} \\ \end{array} } \right.$$

The newly calculated position of addax is denoted as $F_{k}^{m2}$ of $k^{th}$ dimension. The random variable is specified as $G$ and the term $d$ is indicated as the iteration counter. AOA seeks to enhance its capacity to efficiently exploit local areas of the search space by integrating addaxes’ digging talent. The exploration phase based on mimicking addaxes’ habits of foraging is utilized to improve broad search abilities.

Overall, the AOA algorithm’s digging skill offers a distinctive and bio-inspired method of tackling optimization problems by emulating the adaptability and ingenuity of addaxes in their natural environment. The pseudocode of AVOA is offered in Algorithm 1.

**Algorithm 1:**
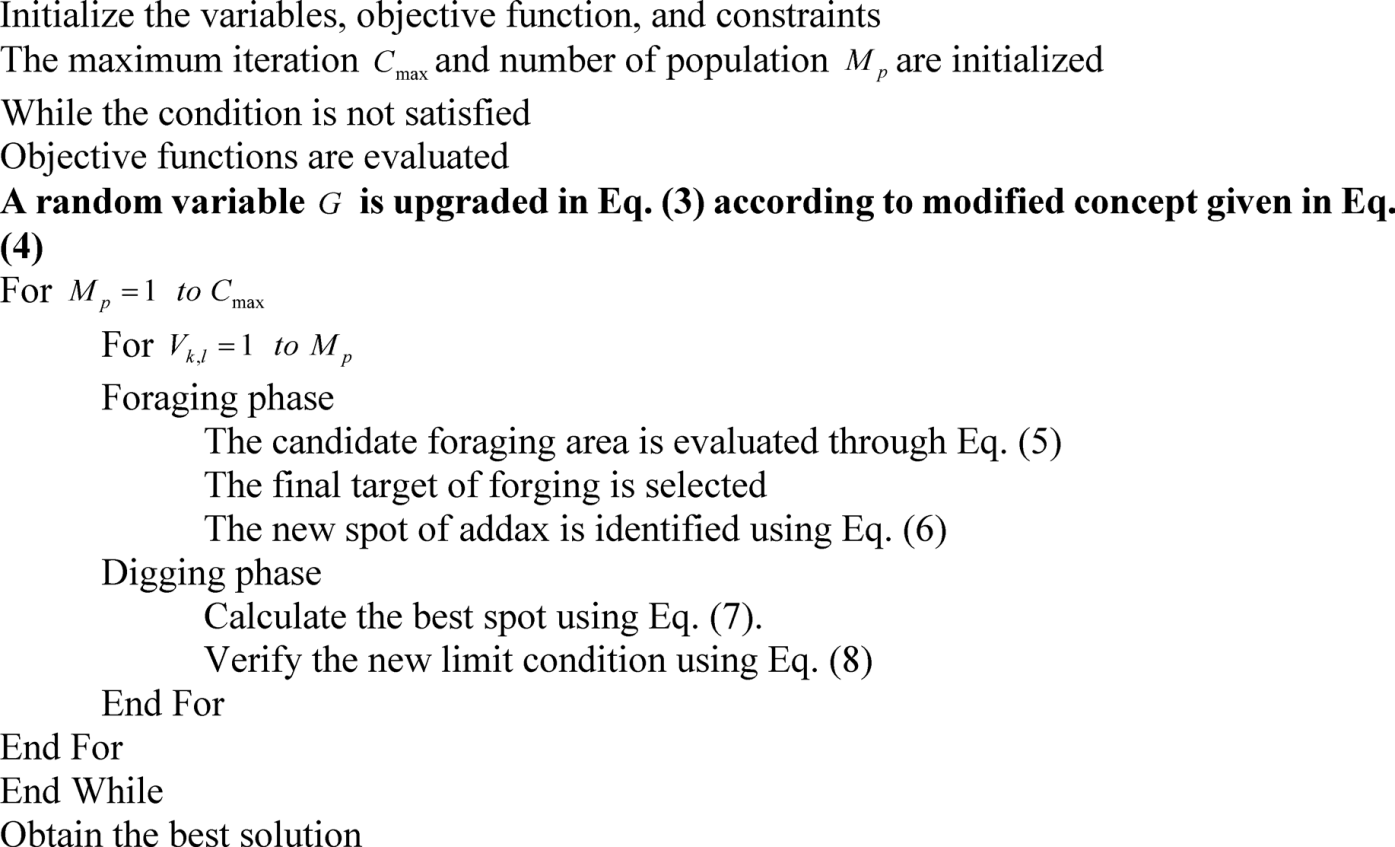
Implemented AVOA

**Fig. 4:**
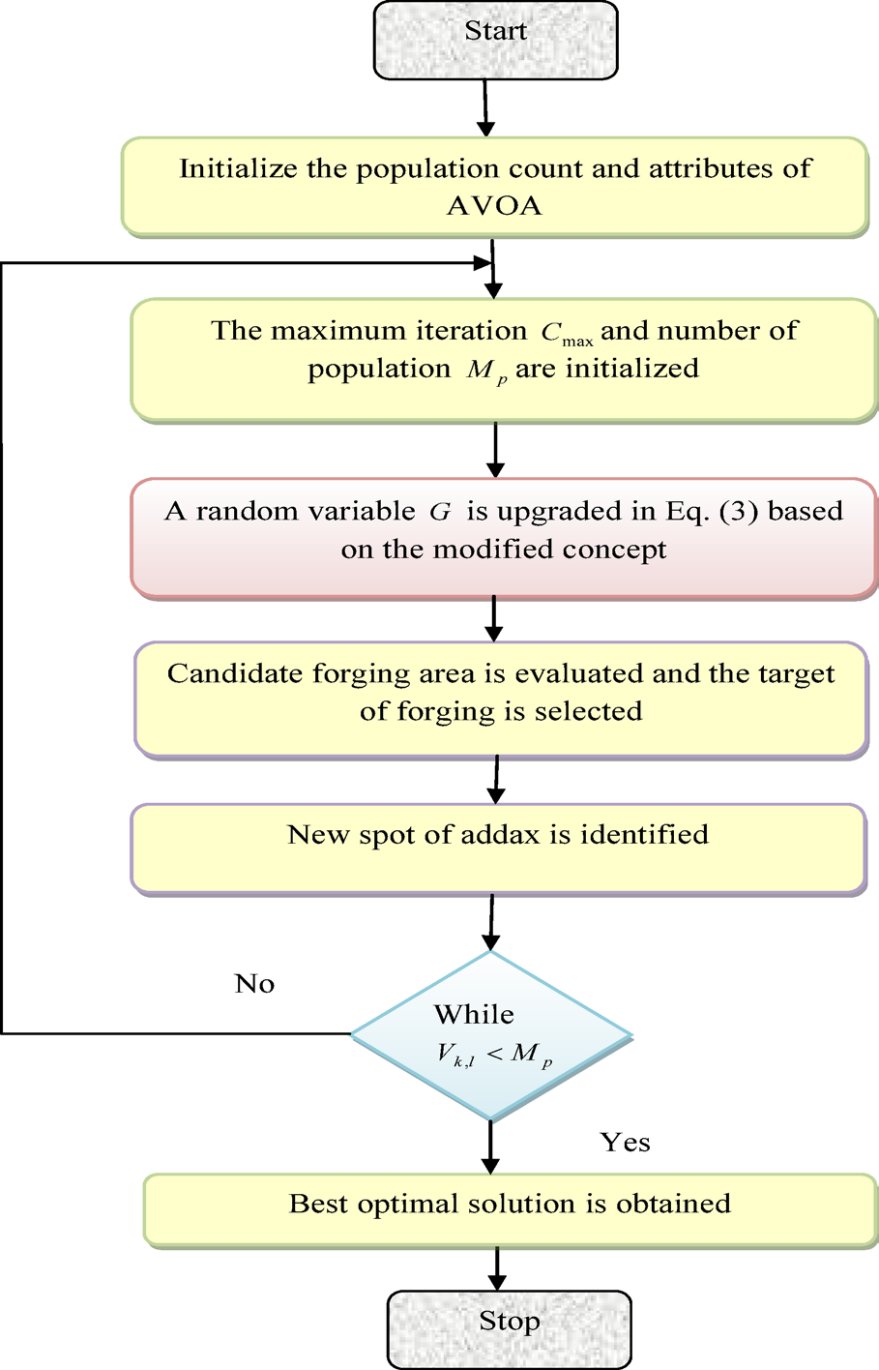
Flowchart of proposed AVOA.

A flowchart of AVOA is provided in Fig. 4.

## Brief elucidation of implemented attention-based deep network for accurate masked face recognition

This section offers deep information about the novel deep learning mechanism employed for recognizing the masked face accurately. Brief information about the efficient attention network is offered in “Efficient attention network description” section. The implementation detail of the newly designed masked face recognition model DS-AEAN is provided in “Implemented DS-AEAN” section. Furthermore, different objectives in DS-AEAN for recognizing the masked faces accurately are detailed in “Objective of masked face recognition using DS-AEAN” section.

### Efficient attention network description

An efficient attention network^32^ is developed in this designed model to reduce the memory and operational challenges of dot-product attention mechanisms. By dropping memory and computational costs, this network upgrades the reliability of performance in many fields such as object detection, instance segmentation, and stereo depth estimation.

Like classic attention mechanisms, the EAN forms queries, keys, and values by linearly transforming input feature vectors. Instead of interpreting the keys as distinct feature vectors for every position, EAN uses them as global attention maps. The formula for describing the mechanism of EAN is provided in Eq. (10).

10 $$R\left( {u,e,a} \right) = \rho_{g} \left( u \right)\rho_{h} \left( e \right)^{y} a$$

Here, the query, key, and value of the matrix are specified as $u$,$e$, and $a$. The normalized function for query and key features are represented as $\rho_{g}$ and $\rho_{h}$. The algebraic forms of normalized functions are specified in Eq. (11) and Eq. (12).

11 $$\rho_{g} \left( u \right) = \frac{u}{\sqrt m }$$

12 $$\rho_{h} (e) = \sigma_{rw} (e),\;and\;\rho_{g} (e) = \sigma_{cl} (e)$$

**Fig. 5:**
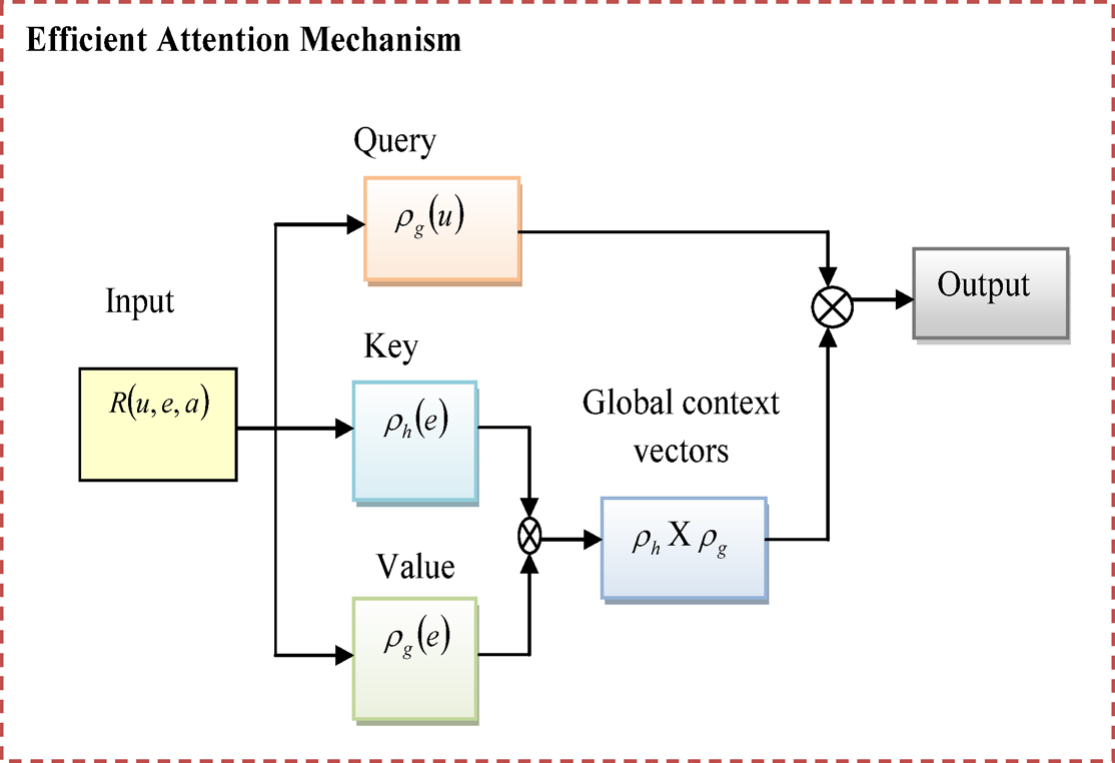
Mechanism of efficient attention network.

The softmax function is applied in queries, keys, and values to generate the output and reliable utilization of resources in the attention mechanism. The basic structural design of an efficient attention network is provided in Fig. 5.

### Implemented DS-AEAN

Recognizing the masked face from the images is a challenging task. Researchers implemented various deep learning and machine learning models for recognizing the masked images. In the classical masked face recognition model, AMaskNet^20^ is prone to lower generalization that may be prone to overfitting, which studies the training information with noise and unwanted details. Collecting inappropriate information for the analysis affects the overall performance of the network. In order to overcome efficiency issues, the developed framework considered the dual scale layers in the masked face recognition network. Including the dual scale layer in the network helps to enhance the overall recognition efficiency in different scales and also maintains the accuracy of recognition in complicated conditions like poor lighting and blurred backgrounds. FaceMaskNet-21^23^ failed to detect the masked face correctly in different face angles and position. Moreover, this technique is not suitable for handling the occlusions in images and also needs enormous memory space for processing the samples. Certain difficulties in this technique aid to more error and misclassification issues. Thus, EAN is suggested in this research work for processing large data sets with minimal memory. The flexibility of the developed framework is improved by learning complicated samples and offering better outcomes in varying conditions. Furthermore, it needs minimal memory space for training the samples. The developed DS-AEAN-based masked face recognition model effectively enhances the final recognition outcomes by considering the local and global patterns, which helps to obtain optimal outcomes than the classical techniques. Using the augmented image for recognizing the masked face helps to improve the generalizability as well as robustness to use in real-world conditions. Moreover, the developed DS-AEAN recognizes the masked face quickly without any errors or misinterpretations.

#### DS-AEAN for handling image quality-based problems

In the developed masked face recognition model, DS-AEAN is employed to execute the feature extraction process. In this phase, DS-AEAN used the GAN-based augmented images as input. Developed DS-AEAN used dual scale, which has the efficiency to execute the validation among small and enormous samples in various scales. Moreover, using a dual scale helps to process the higher and lower quality samples and it easily selects the higher quality information required for the validation. It processes all types of image samples and aids in offering better outcomes without affecting the final outcomes. Moreover, the dual scale layer enhances the overall flexibility of the network that makes the developed framework to suitable for all conditions. So, adding the dual scale layer in EFN offers more advantages to handle all types of images. In the developed DS-AEAN, higher quality and blurred quality images are considered as the input and the developed DS-AEAN offered more accurate masked face-recognized outcomes. The developed DS-AEAN outcomes didn’t depend upon the quality of the image. Moreover, it offers better outcomes by considering the EAN, which has higher interpretability in observing the most relevant feature. Thus, this section concluded that the developed DS-AEAN is capable of processing all kinds of images without affecting the overall performance of the network.

**Fig. 6:**
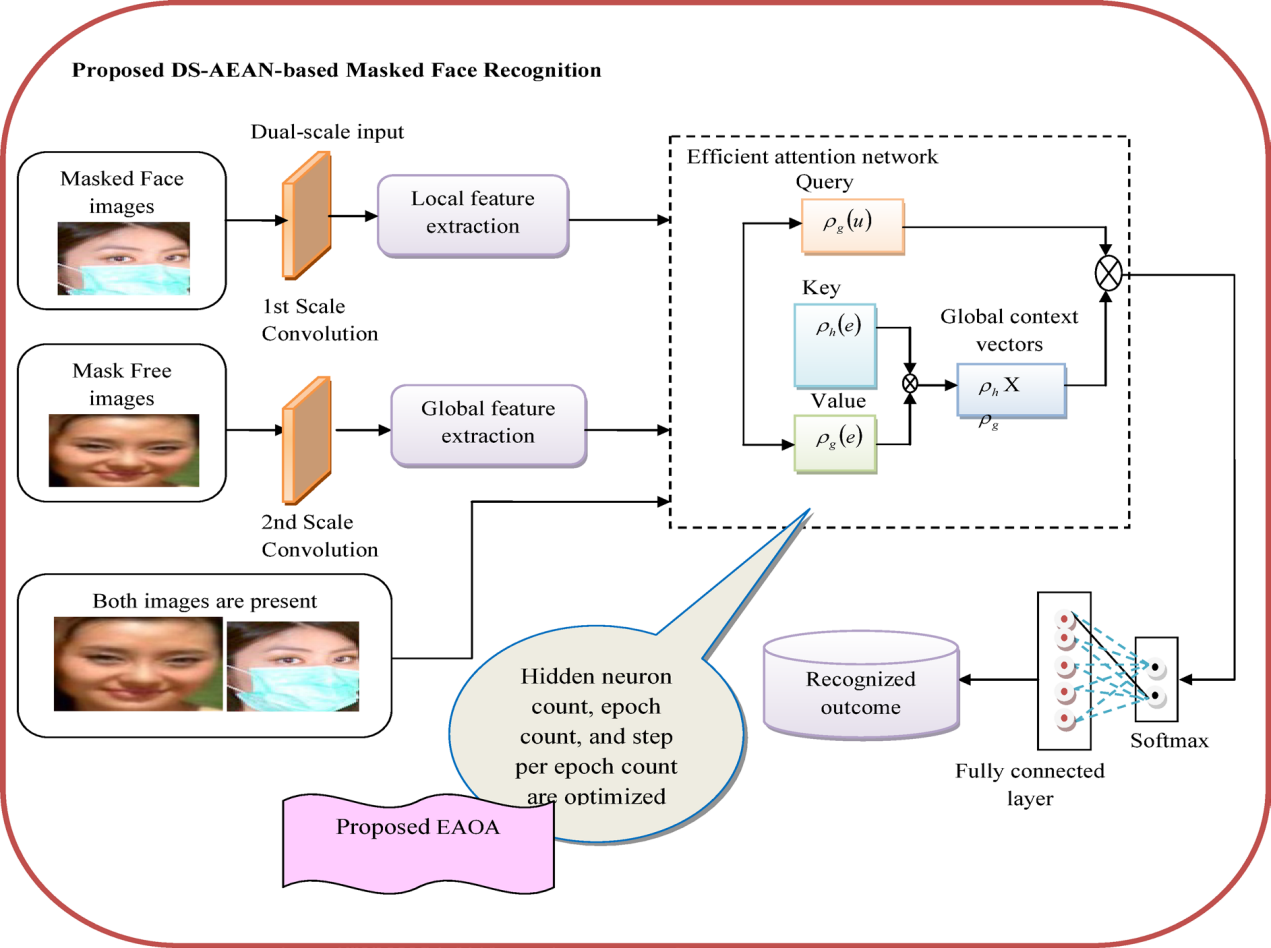
Structural illustration of DS-AEAN model.

GAN-based augmented images are applied to DS-AEAN for the recognition of masked faces. If the input images contain both masked and mask-free images, then it is directly given to DS-AEAN for categorization purposes. Designed DS-AEAN integrates dual-scale extracted features and an adaptive attention mechanism for masked facial recognition tasks. Using this dual-scale attention layer, the designed DS-AEAN focuses on the distinct size of features from the input data. By focusing on both local and global features at the same time, this network captures more extensive and useful representations resulting in greater efficiency in extensive feature extraction. Selective feature fusion is performed by highlighting important features at various scales. This selective fusion method enables the model to efficiently incorporate information from various scales in order to make more informed decisions, hence increasing the network’s total descriptive capacity. The improved spatial context modelling enables us to comprehend the spatial layout of features and objects in input images, resulting in more precise and context-aware predictions. The attention mechanism modifies the relevance of features at distinct scales in response to task needs. This adaptability enables the network to focus on the most suitable data for a specific job, hence enhancing the ability of the model to extract meaningful characteristics and generate better predictions. DS-AEAN efficiently minimizes its computational burden by focusing on certain scales of features. By focusing on key details and removing irrelevant data, this network speeds up the process of acquiring features and optimizes computing resources, resulting in more efficient model training and inference. Dual-scale attention enables the model to accommodate scale variations in input data, such as objects of varying sizes or resolutions. By focusing on characteristics at several scales, the network adapts to changing object sizes while maintaining consistent performance across a wide range of input conditions and boosting the reliability of the masked facial recognition model. The structural illustration of the DS-AEAN model is provided in Fig. 6.

### Objective of masked face recognition using DS-AEAN

Masked face recognition with a dual-scale attention mechanism involves exploiting the strengths of attention mechanisms at several scales to improve recognition accuracy, particularly in cases when faces are partially covered by masks. By focusing on features of multiple sizes, the dual-scale attention mechanism allows the model to derive deeper and more informative depictions of masked faces. If the input images contain both masked and mask-free images, then it is directly given to DS-AEAN for categorization purposes. This improved description of features helps the model to discriminate between persons based on observable face features, even with a mask. The dual-scale attention mechanism assigns adaptive attention weights to several scales of features, allowing the simulator to dynamically modify the relevance of information dependent on the context of the masked face. This adaptability allows the framework to focus on key facial regions while suppressing unwanted distractions induced by the presence of masks. Masked face recognition frequently requires dealing with occlusions induced by masks, which hamper typical recognition methods. This dual-scale attention method improves the model occlusion robustness by highlighting crucial face characteristics that remain visible in the presence of masks, resulting in higher identification accuracy in difficult settings. It generates interpretable attention maps that emphasize regions of interest on various scales. This interpretability assists users in understanding how the model processes masked images of faces and which facial regions have the greatest impact on the recognition decision. The designed DS-AEAN model improves its visibility and confidence by optimizing the parameters like hidden neuron count, step per epoch count and epoch count from DS-AEAN through EAOA, which maximizes the accuracy of the recognition model. The objective function is mathematically provided in Eq. (13).

13 $$FR_{obj} = \mathop {\arg \min }\limits_{{\left\{ {Fc_{g}^{DS - AEAN} ,Hj_{m}^{DS - AEAN} ,Th_{m}^{DS - AEAN} } \right\}}} \left( \frac{1}{Ay} \right)$$

Here, the term $Fc_{g}^{DS - AEAN}$ specifies hidden neuron count at the range of $\left[ {5 - 225} \right]$, $Hj_{m}^{DS - AEAN}$ denotes epoch count in the limit $\left[ {5 - 50} \right]$, and $Th_{m}^{DS - AEAN}$ signifies step per epoch count in the bound $\left[ {5 - 250} \right]$. The aim of optimization is the maximization of accuracy $Ay$. Moreover, accuracy is provided in Eq. (14).

14 $$Ay = \frac{{Q^{ * } + R^{ * } }}{{Q^{ * } + R^{ * } + H^{ * } + K^{ * } }}$$

Here, the true positive and negative values are specified as $Q^{ * }$ and $R^{ * }$ respectively. The false positive and negative value is specified as $H^{ * }$ and $K^{ * }$, correspondingly.

## Results and discussions

In this section, various experiments are executed to verify the performance of the developed masked face recognition model over existing models. Different recognition works employed for the validations and also parameter setting details are offered in “Experimental analysis” section. Multiple performance measures used in the validation and their mathematical representation are offered in “Resultant Outcome using GAN” section. The rest of the section provides detailed observations on the developed masked face recognition model over classical techniques.

### Experimental analysis

The experimental analysis of a masked face identification model involves studying the behavior of the model during training and implementation, which was done using Python software. This model was trained with a number of population as 10, a maximum iteration as 50 and a chromosome length as 3 to compute the reliability of the masked face recognition model. By conducting experiments within the setup, researchers optimize the model attributes, fine-tune the models, and upgrade the overall performance of the system. This optimization process leads to a more successful and effective masked face recognition. Several conventional algorithms like the Reptile Search Optimizer (RSA)^33^, Tomtit Flock Metaheuristic Optimization Algorithm (TFMOA)^34^, Golf Optimization Algorithm (GOA)^35^, and Addax Optimization Algorithm (AOA)^31^ were compared to find out the most effective approach for masked face recognition. Some of the existing classifiers like Long Short-Term Memory (LSTM)^36^, ResNet^37^, InceptionNet^38^, and Efficientnet^39^ were also compared to select the optimal model for deployment. Conventional classifiers like AMaskNet^20^, DeepMaskNet^21^, FaceMaskNet-21^23^ and MobileNetV2^25^ were also employed to verify the efficiency of the developed framework. Information about model size and memory requirements is offered in Table 2.

**Table 2:** Details about model size and memory requirements.

Techniques	Memory requirements (MB)	Model size (Millions)
RSA^33^	500	–
TFMOA^34^	750	–
GOA^35^	420	–
AOA^31^	650	–
Proposed EAOA	300	–
LSTM^36^	650	0.3
Resnet^37^	910	25.6
InceptionNet^38^	710	42.7
Efficientnet^39^	680	66
Proposed DS-AEAN	300	50

### Resultant outcome using GAN

**Fig. 7:**
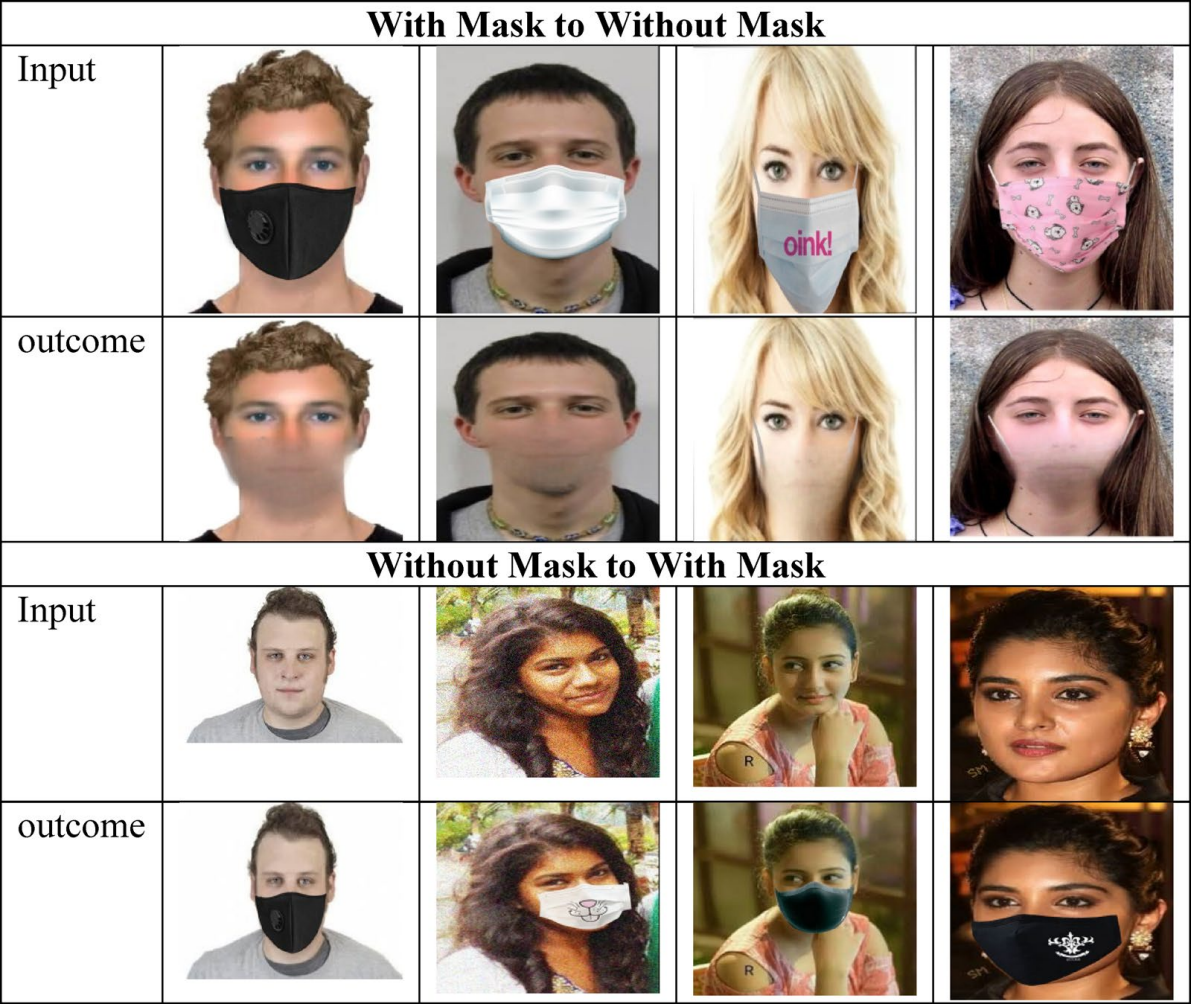
Resultant image outcome using GAN.

GAN is trained in an aggressive way to produce high-quality synthetic data. It is capable to generating both mask-free images and masked face images as outcome. The resultant image outcome is provided in Fig. 7.

### Validating cost function of proposed framework

**Fig. 8:**
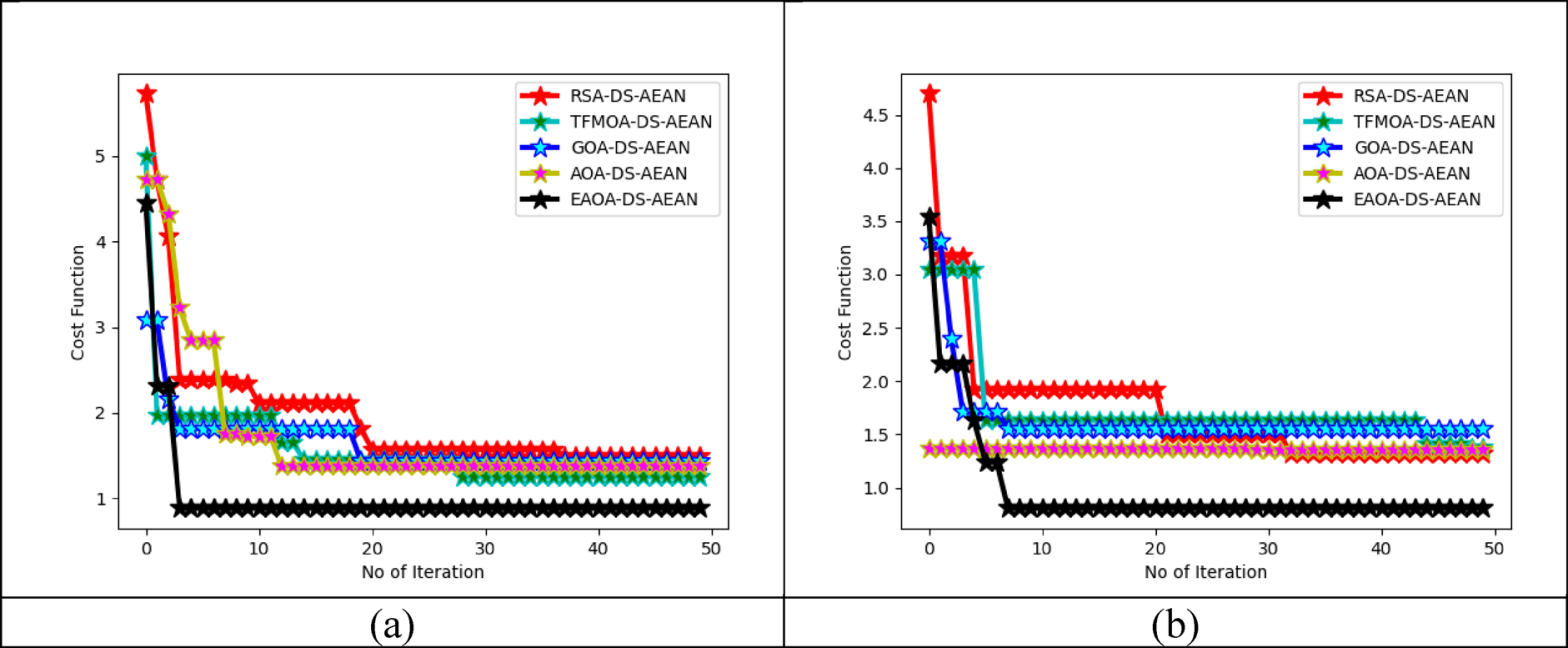
Cost function validation on recommended masked face recognition technique among dataset-1 and dataset-2.

Cost function analysis is performed to evaluate the performance of the proposed masked face recognition system under various conditions and scenarios. This helps in assessing the success rate of the system in real-world applications. The graphical view of cost function analysis is provided in Fig. 8. Convergence analysis of a masked face recognition model involves studying the behavior of the model during training to ensure that it converges to a stable solution. During the training process, the learning rate is analyzed and helps the model to converge faster and reach a better optimum solution. The learning rate is appropriately adjusted to prevent overfitting issues. The cost function of the proposed model helps to prevent overfitting and boost the convergence behavior of the model by promoting smoother optimization and generating better efficiency in the masked face recognition model.

### Computation of ROC curve

**Fig. 9:**
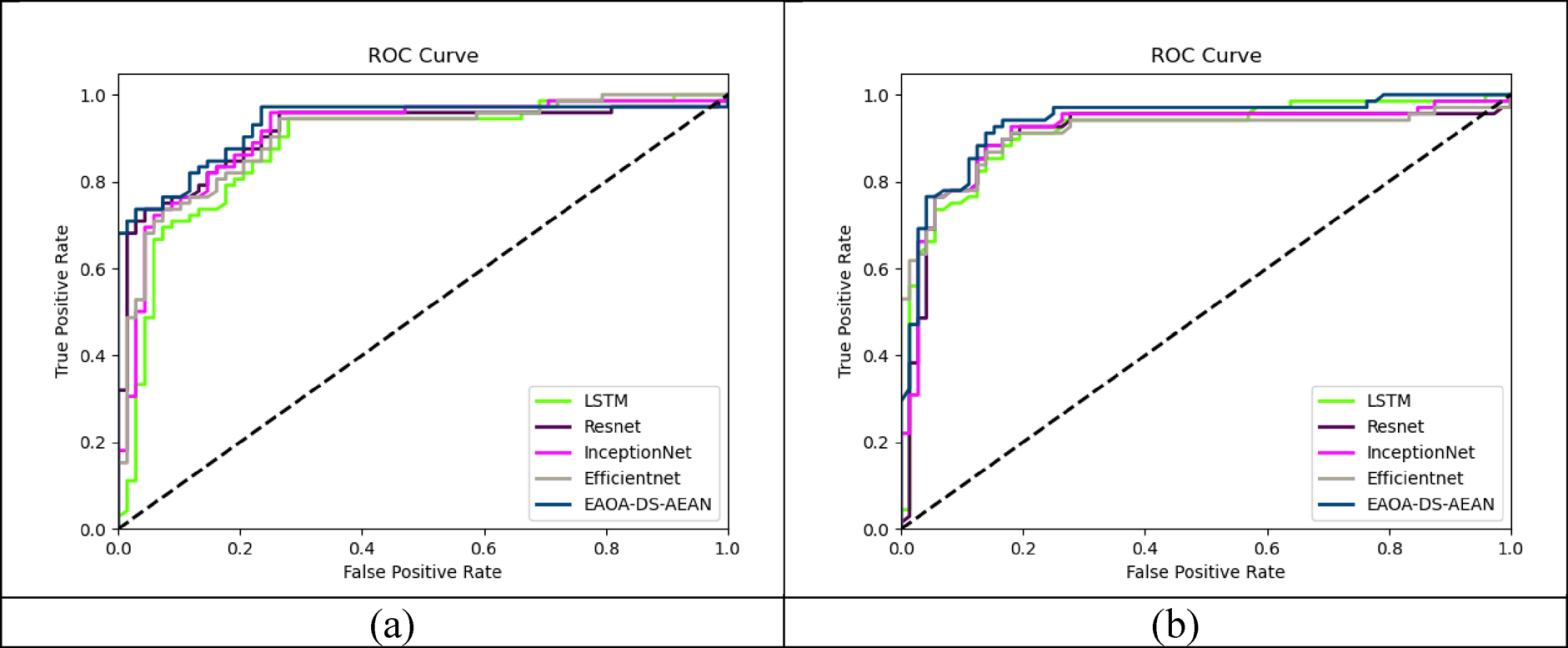
ROC curve computation on developed masked face recognition technique among (**a**) Dataset 1, and (**b**) Dataset 2.

ROC curve validation offers an effective way to evaluate the reliability of the designed framework by plotting the true positive values over false positive values with threshold adjustments. This analysis helps in assessing the system’s capability to distinguish between different classes, such as masked and unmasked faces. The ROC curve among both dataset 1 and dataset 2 are plotted in Fig. 9 to provide a visual representation of system performance. ROC curve analysis provides the comparison of different face recognition classifiers like LSTM, ResNet, InceptionNet, and EfficientNet which helps in identifying the most effective classifier for masked face recognition tasks. By analyzing the ROC curve, the robustness of the face identification system to variations in the dataset is assessed to identify the masked individuals and minimize false identifications.

### K-fold cross-validation on developed technique over existing recognition techniques

**Fig. 10:**
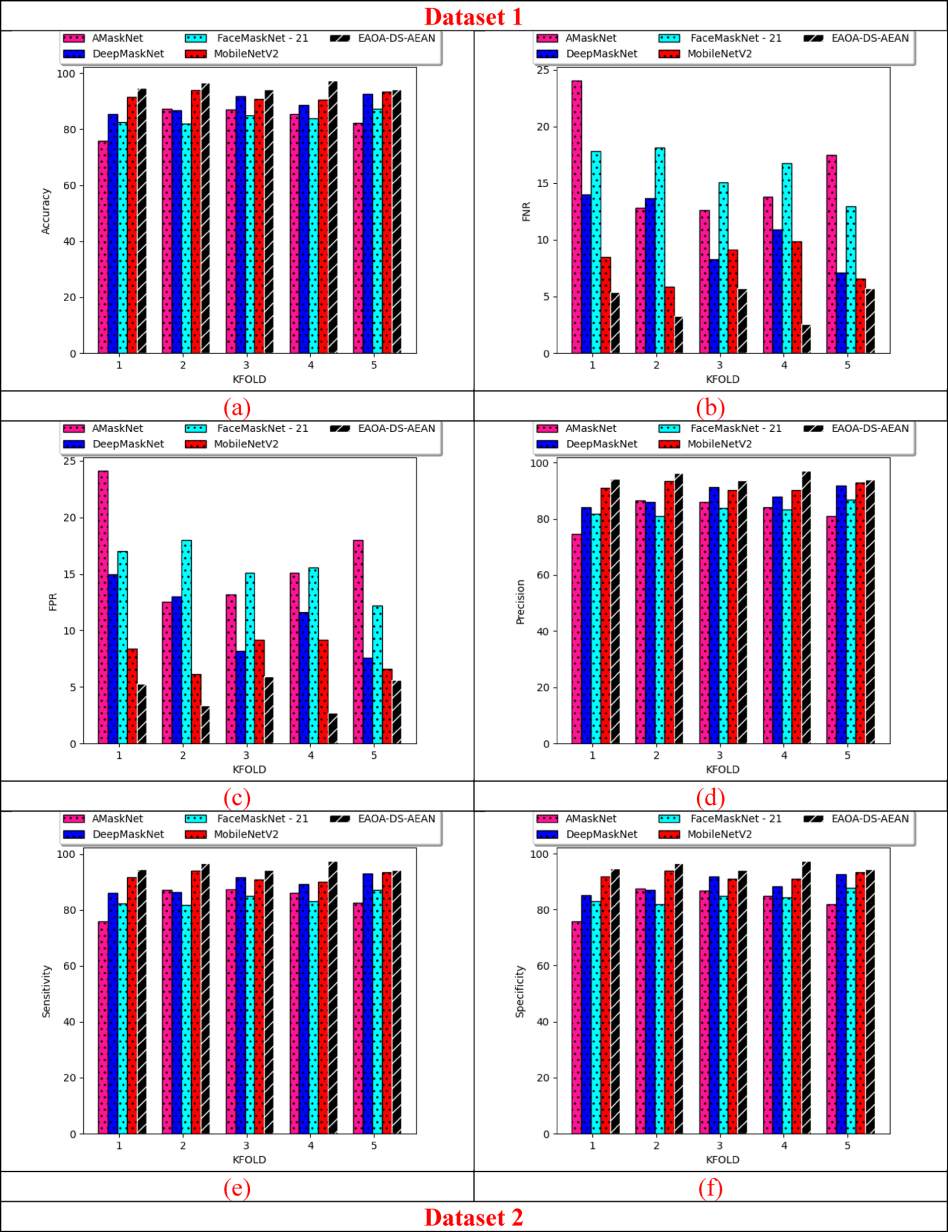

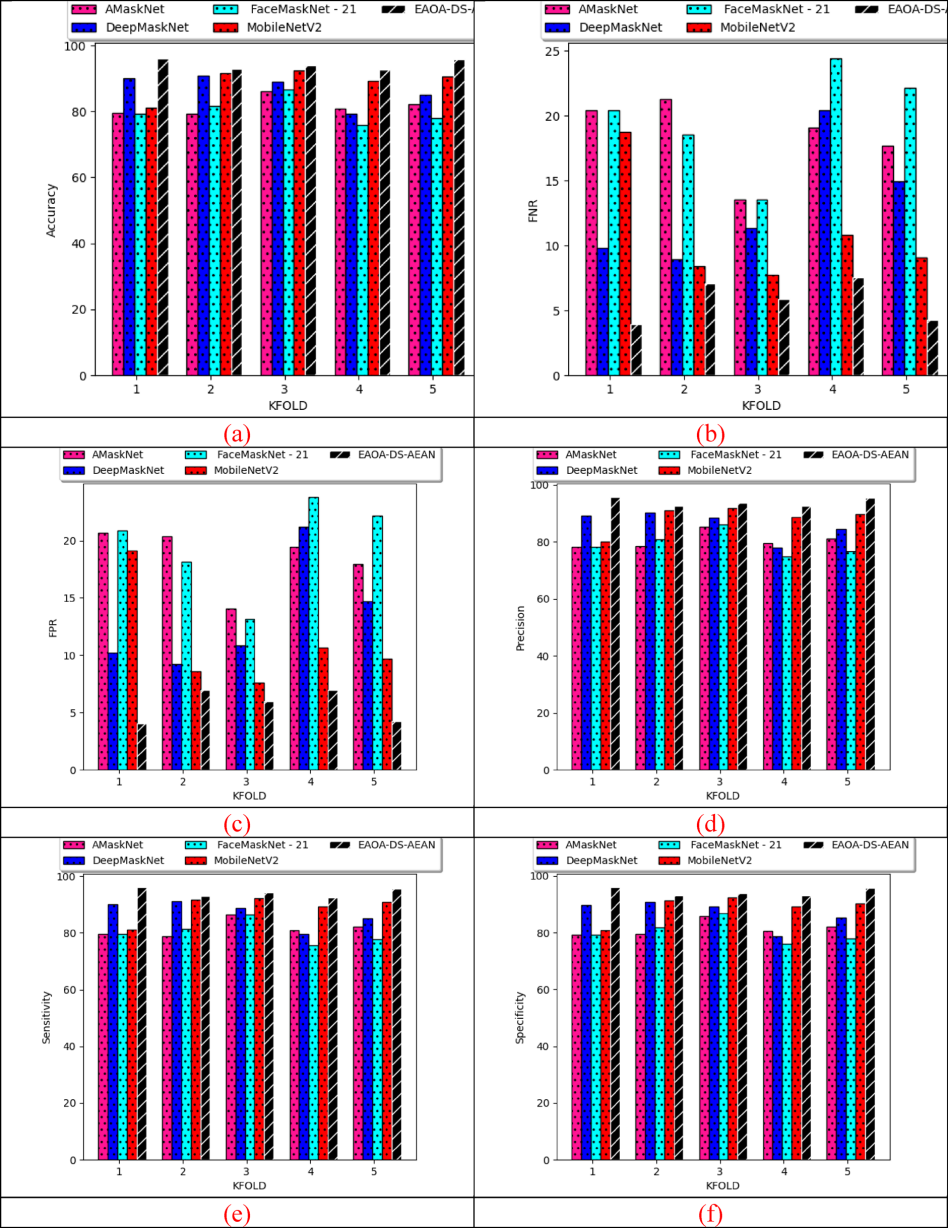
Effectiveness analysis of the implemented masked face recognition model among classical recognition techniques according to (**a**) Accuracy (**b**) FNR (**c**) FPR (**d**) precision (**e**) sensitivity and (**f**) specificity for both datasets.

K-fold assessments on the suggested framework over multiple techniques for both datasets are specified in Fig. 10. K-fold cross-validation helps to avoid overfitting in the model while training the data based on each fold. This helps to ensure that the designed framework is efficient in different folds, including masked face recognition. K-fold cross-validation helps in reducing bias in reliability estimation minimizes the impact of data partitioning on performance evaluation and provides a more unbiased assessment. From this analysis, the precision rate of the proposed model is improved as 23.07% than AMaskNet, 14.2% than DeepMaskNet, 20% than FaceMaskNet-21, and 9.9% than MobileNetV2 at K-fold value as 1 for dataset 1. Thus, the benefits of K-fold cross-validation analysis in face recognition systems are providing robust performance estimation, optimising parameter tuning, reducing overfitting, maximising data utilization, reducing bias, and helping in the appropriate selection of a classifier.

### K-fold cross-validation of the proposed model over algorithms

**Fig. 11:**
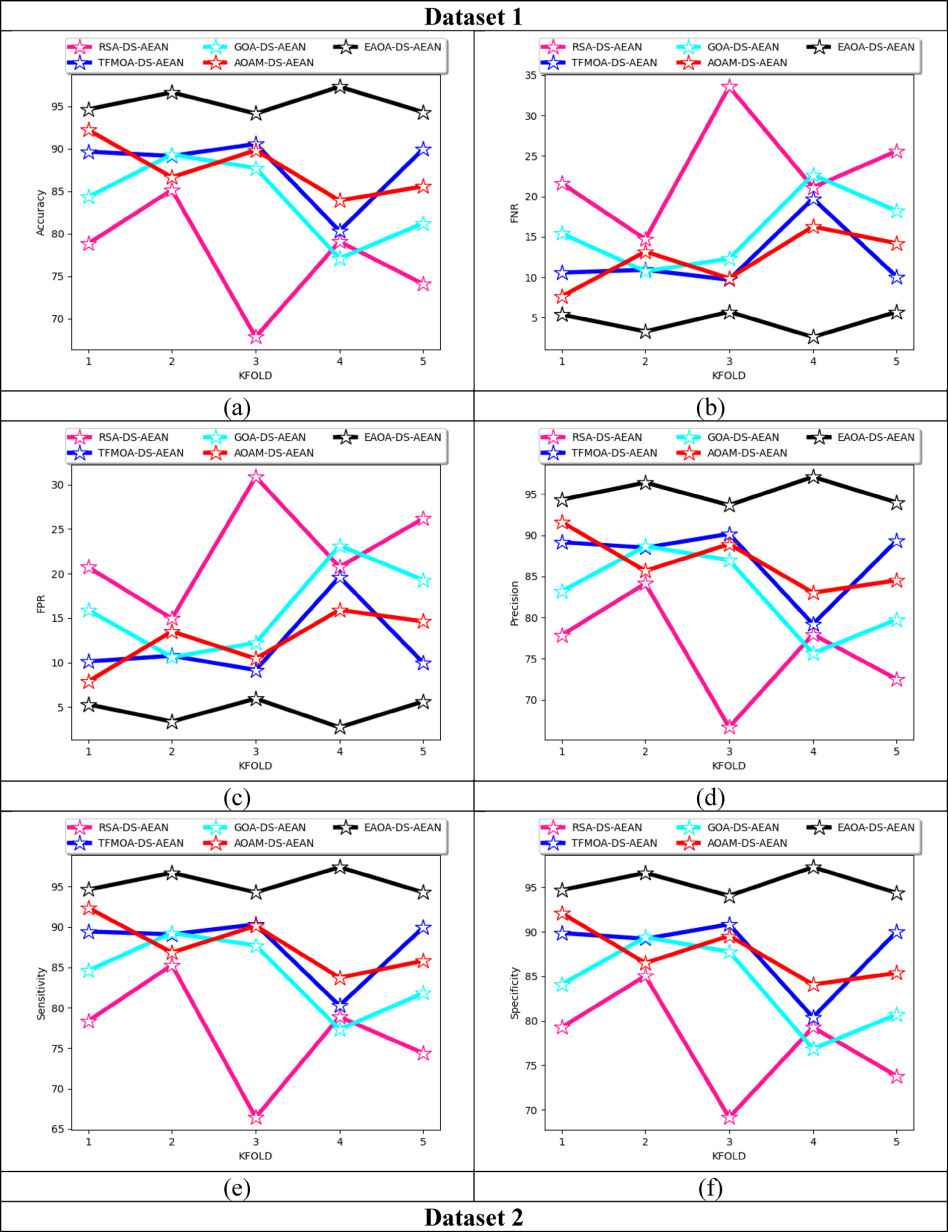

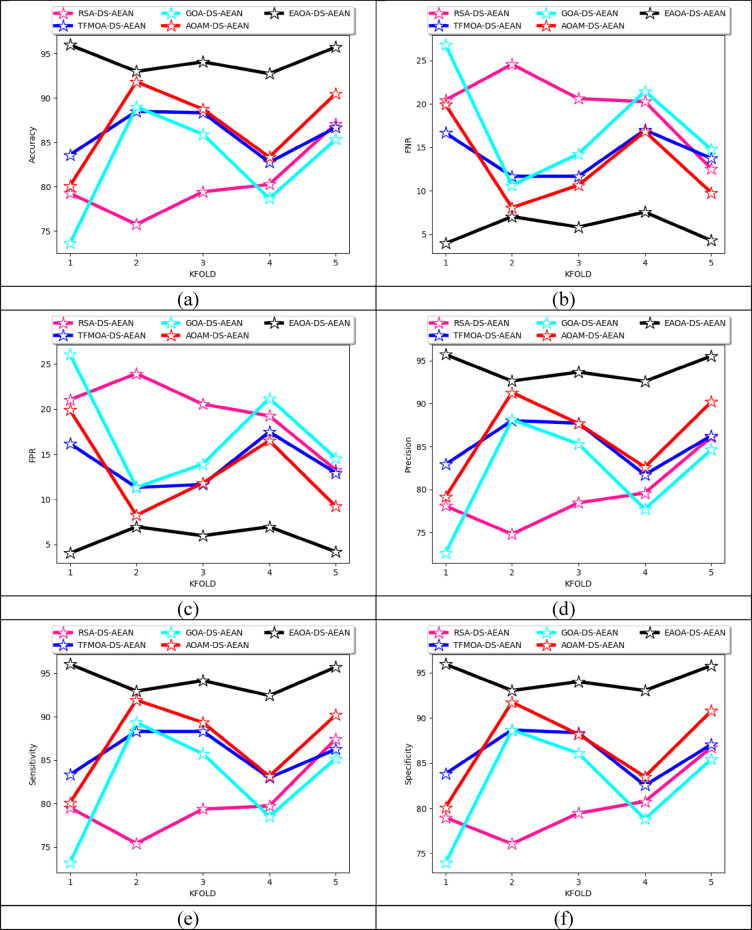
Effectiveness analysis of the implemented deep learning-based proposed masked face recognition model among various algorithms according to (**a**) Accuracy (**b**) FNR (**c**) FPR (**d**) precision (**e**) sensitivity and (**f**) specificity for both datasets.

K-fold assessments of recommended masked face recognition techniques over various algorithms for both datasets are specified in Fig. 11. During the training phase, the dataset is divided into numerous k-folds for maintaining class balance. Train the designed model based on different algorithms on the training set and evaluate their evaluations on the validation set. Compute the performance of the designed model using metrics like accuracy, FNR, FPR, precision, sensitivity, and specificity for each fold. Based on dataset 2, the specificity of the designed model is enhanced with 16.4% than RSA-DS-AEAN, 9.5% than TFMOA-DS-AEAN, 26.02% than GOA-DS-AEAN, and 15% than AOAM-DS-AEAN. K-fold assessment helps in evaluating the model success rate and comparing it with established algorithms. It provides maximum utilization of available data for both training and testing purposes. Thus, the strength of the designed classifier is boosted by reducing the impact of data variability and overfitting issues for better recognition.

### Statistical analysis

The numerical value of the statistical computation on the recommended technique is given in Table 3. Generally, statistical computations are executed over 50 iterations, with the consideration of several objectives offered in Eq. (13). Statistical validations are widely performed by considering worst, best, mean, median, and standard deviation, which helps to offer better-masked face recognition outcome. Higher masked face-recognized values obtained over entire iterations are termed as best. In real-world usage, the best value helps to offer better decision making. Attained minimum values over iterations are considered as worst, which displayed the poor efficiency of the prediction network. This validation helps to obtain more accurate masked face recognition outcomes through EAOA-DS-AEAN. Moreover, better-masked face recognition outcomes are accomplished by EAOA-DS-AEAN while the objective accuracy is fulfilled. It helps to assess the efficient strategy to mitigate the potential hazard during training. Using dataset 2, the best value of the suggested technique is upgraded with 39.1% than RSA-DS-AEAN, 41.6% than TFMOA-DS-AEAN, 48.2% than GOA-DS-AEAN, and 40.6% than AOAM-DS-AEAN. By incorporating statistical analysis techniques, researchers can rigorously evaluate the performance of the designed model, assess its authority over conventional algorithms, and provide robust verification to support their research findings in the domain of masked facial recognition.

**Table 3:** Statistical analysis of the proposed masked face recognition technique over different algorithms.

Statistical Measures	RSA-DS-AEAN^33^	TFMOA-DS-AEAN^34^	GOA-DS-AEAN^35^	AOA-DS-AEAN^31^	EAOA-DS-AEAN
For Dataset 1					
Mean	1.966034	1.555609	1.640998	1.731638	1.013628
Standard deviation	0.811465	0.56227	0.350447	0.839157	0.565627
Worst	5.723826	4.990873	3.077338	4.720293	4.454418
Median	1.572208	1.447998	1.440467	1.378789	0.885091
Best	1.502703	1.255088	1.440467	1.378789	0.885091
For Dataset 2					
Worst	4.69854	3.04118	3.307179	1.362483	3.546474
Standard deviation	0.621178	0.438882	0.359072	0.003345	0.503078
Median	1.505841	1.628714	1.555422	1.362483	0.80441
Best	1.322905	1.378179	1.555422	1.355706	0.80441
Mean	1.747463	1.742884	1.654678	1.359637	0.974629

### Numerical analysis of the proposed model by varying epoch count

Analyzing the epoch count helps in assessing how quickly the model converges to the best solution during training. Understanding the convergence behavior provides quick training. The value of accuracy is analyzed among different algorithms and techniques by varying epoch counts are provided in Tables 4 and 5. Determining the suitable number of epochs helps in optimizing training time and computational resources, particularly in scenarios where training large models on extensive datasets is a time-consuming process. At the 300^th^ epoch count, the reliability of the designed framework is upgraded with 49.1% than RSA-DS-AEAN, 12.04% than TFMOA-DS-AEAN, 24.3% than GOA-DS-AEAN, and 13.4% than AOAM-DS-AEAN for dataset 1. Epoch count analysis helps in defining early stopping criteria based on validation performance, protecting the model from overfitting, and reducing the utilization of energy by stopping training at the right time. Thus, by performing epoch count analysis, researchers will fine-tune their deep learning models, upgrade the efficacy of training, and ultimately achieve better performance on various masked face recognition tasks.

**Table 4:** Accuracy analysis of proposed masked face recognition technique by varying epoch count among various algorithms.

Epoch count	RSA-DS-AEAN^33^	TFMOA-DS-AEAN^34^	GOA-DS-AEAN^35^	AOA-DS-AEAN^31^	EAOA-DS-AEAN
For dataset 1					
100	72.75	81.75	76.66666667	85.83333333	95.83333333
200	70.58333333	89.25	82.91666667	90.33333333	96.83333333
300	65	86.5	77.91666667	85.41666667	96.9166666
400	66.33333333	80.83333333	84.83333333	89.16666667	96.33333333
500	71	82	85.58333333	91.08333333	96.83333333
For dataset 2					
100	74.75	80.91666667	81.66666667	84.41666667	94.25
200	74.66666667	90.83333333	89.08333333	85.83333333	93.83333333
300	86.5	80.25	74.75	87	92.33333333
400	77.41666667	78.58333333	83.91666667	88.5	95.91666667
500	85.33333333	86.25	80.75	83.75	95

**Table 5:** Accuracy validation of the proposed masked face recognition technique by varying epoch count over multiple recognition models.

Epoch count	LSTM^36^	Resnet^37^	InceptionNet^38^	Efficientnet^39^	EAOA-DS-AEAN
For dataset 1					
100	84.75	89.5	82.41666667	85.25	95.83333333
200	88.16666667	84.33333333	82.75	92.83333333	96.83333333
300	87.25	85.66666667	86.08333333	92.08333333	96.91666667
400	82.91666667	84.83333333	80.41666667	85.83333333	96.33333333
500	89.33333333	86.41666667	83.83333333	94.16666667	96.83333333
For dataset 2					
100	86	88.41666667	86.41666667	81.41666667	94.25
200	86.75	81.66666667	80.66666667	92.33333333	93.83333333
300	87.08333333	90.25	78	89.16666667	92.33333333
400	83.58333333	85.83333333	75.91666667	85.83333333	95.91666667
500	76.91666667	83.41666667	79.08333333	87.83333333	95

### Validation on execution time

The execution time of the developed masked face recognition model and the existing technique is displayed in Table 6. In this phase, the InceptionNet^32^ gained a higher execution time at 40.48 min than other techniques. Attaining a higher execution time in the masked face recognition model affects the overall performance and also causes several delays in obtaining better outcomes. Furthermore, the analysis outcome displayed that the recommended framework gained a minimal execution time as 33.12 min than the classical technique. Accomplishing minimal execution time helps to improve the overall response and also enhances the masked face recognition process.

**Table 6:** Observation on execution time in developed framework.

Comparison	Techniques	Execution time (mins)
Optimization models	RSA^33^	36.13
	TFMOA^34^	38.45
	GOA^35^	39.02
	AOA^31^	37.32
	Proposed EAOA	33.12
Face recognition techniques	LSTM^36^	39.52
	Resnet^37^	35.19
	InceptionNet^38^	40.48
	Efficientnet^39^	38.23
	Proposed DS-AEAN	33.12

### Masked face recognition model in real-world application scenarios

Generally, masked face recognition techniques are employed to identify the individuals roaming in public places without masks. The masked face recognition techniques are widely applicable in various real-world applications and they are detailed as follows. Masked face recognition models gained more attention from healthcare industries. This technique helps to identify the individual who comes for medical procedures and normal checkups. In the transportation field, it aids in identifying the passengers at the boarding gates and security checking points. In order to enhance the security and access control in the restricted regions and sensitive locations masked face recognition models are more useful. Most of the multi-national organizations employ the masked face recognition technique to track the presence of employees and also to monitor the employees in specific work regions. Moreover, this technique helps to ensure the safety requirements of individuals in crowded public spots to avoid criminal activities. In some cases, these frameworks are widely suggested in the biometric security system by considering specific features from individuals faces. Thus, it is concluded that the masked face recognition models are widely suitable in different real-world scenarios for monitoring individuals in huge gatherings and other conditions.

## Conclusion

An efficient masked face recognition model was developed for accurately identifying individuals even when wearing masks, this model upgraded the security measures in access control systems, and identity verification systems. The face image with mask and without masks was gathered from the organized database and fed to the GAN-based augmentation phase for extracting the robust features and boosting their performance in the categorization phase. The augmented image was applied to DS-AEAN for the recognition of the face. Dual-scale networks help in localizing objects within an image more precisely by combining information from different scales. The exactness of this designed model was boosted by fine-tuning the attributes from DS-AEAN using EAOA to generate a highly accurate recognized face image. Optimization strategy helps in efficiently utilizing computational resources such as memory, processing power, and storage, making models more scalable and cost-effective to perform recognition tasks. This designed model enhances the efficiency of security measures by identifying individuals even when wearing masks, aiding in surveillance and access control by reducing error. The success rate of the designed model was enhanced with 9.5% than LSTM, 6.5% than ResNet, 9.06% than InceptionNet, and 15.7% than EfficientNet at the 100^th^ epoch count using dataset 2. Thus, the designed model improves security measures by accurately recognizing individuals even when wearing masks, reducing the risk of unauthorized access, and boosting surveillance capabilities in susceptible areas such as airports, government buildings, and financial institutions.

### Future work

Integrating multimodal data sources, such as thermal imaging and depth sensing will upgrade the ability of the designed model to detect masked faces and improve recognition accuracy in complex scenarios. Moreover, the validation of the proposed model’s efficiency in practical situations will be considered in future work.

## Declarations

### Data availability

Dataset 1: The data underlying this article are available in “https://www.kaggle.com/datasets/omkargurav/face-mask-dataset” accessed on 2024-06-28. Dataset 2: The data underlying this article are available in “https://github.com/X-zhangyang/Real-World-Masked-Face-Dataset/tree/master” accessed on 2024-06-27.
